# LncRNA-MALAT1 Regulates Cancer Glucose Metabolism in Prostate Cancer via MYBL2/mTOR Axis

**DOI:** 10.1155/2022/8693259

**Published:** 2022-05-02

**Authors:** Xiangyu Mu, Zhou Shen, Yao Lin, Jun Xiao, Kaiguo Xia, Congyun Xu, Biao Chen, Ronghua Shi, Anyang Zhu, Xinzhe Sun, Tao Tao, Xiaoyuan Song, Qiang Xuan

**Affiliations:** ^1^Department of Urology, Anhui Provincial Hospital, The First Affiliated Hospital of USTC, Division of Life Sciences and Medicine, University of Science & Technology of China (USTC), Hefei, Anhui 230001, China; ^2^Department of Urology, The First Affiliated Hospital of Anhui Medical University, Hefei 230022, China; ^3^MOE Key Laboratory for Cellular Dynamics, CAS Key Laboratory of Brain Function and Disease, School of Life Sciences, Division of Life Sciences and Medicine, University of Science and Technology of China, Hefei, Anhui 230026, China

## Abstract

It is known that the long noncoding RNAs (lncRNA) MALAT1 is associated with tumorigenesis and progression in various cancers; however, its functions and mechanisms in prostate cancer (PCa) initiation and progression are still unknown. In the present study, our findings revealed that MALAT1 plays a critical part in regulating PCa proliferation and glucose metabolism. Knockdown of MALAT1 affects the protein and mRNA levels of MYBL2. In addition, MALAT1 enhances the phosphorylation level of mTOR pathway by upregulating MYBL2. Knockdown of MALAT1 or MYBL2 in PCa cell lines significantly inhibits their proliferation capacity. Silencing MALAT1/MYBL2/mTOR axis in PCa cell lines affects their glycolysis and lactate levels, and we verified these findings in mice. Furthermore, we explored the underlying tumorigenesis functions of MYBL2 in PCa and found that high expression of MYBL2 was positively associated with TNM stage, Gleason score, PSA level, and poor survival rate in PCa patients. Taken together, our research suggests that MALAT1 controls cancer glucose metabolism and progression by upregulating MYBL2-mTOR axis.

## 1. Introduction

Prostate cancer (PCa) is the second most common malignant tumor in males of Western countries, but its five-year survival rate (98%) is much higher than that of renal cell carcinoma (75%) and bladder cancer (77%) [1]. However, due to the lack of obvious clinical symptoms in early PCa, most patients were in the middle and late stage at the time of discovery and missed the optimal treatment time [2]. Metastasis-associated lung adenocarcinoma transcript 1 (MALAT1) is a long noncoding RNA (lncRNA) highly expressed in several cancers types and is associated with many aspects of cancer progression [3]. MALAT1 plays a critical role in tumorigenesis and progression of some cancers and has been considered a potential target in CRPC treatment [4, 5]. Although the function of MALAT1 has been widely reported in cancer, the underlying mechanisms in PCa are yet to be elucidated.

MYB proto-oncogene like 2 (MYBL2) belongs to the MYB transcription factor family and plays a critical role in regulating cell proliferation and apoptosis [6, 7]. Various types of cancer reveal that the overexpression of MYBL2 results in aggressive tumors and a low survival rate [8–10]. In PCa, MYBL2 controls the development of castration resistance and metastatic relapse through Hippo-YAP signaling pathway [11]. Since MYBL2 is a potentially carcinogenic gene, its role in tumorigenesis and progression, as well as the mechanisms and downstream signaling pathways, has been under intensive investigation. Interestingly, the classical mTOR signaling pathway controls tumorigenesis and progression [12], and a potential correlation between MYBL2 and mTOR has been detected in *Arabidopsis* [13] via the GSK3b pathway. Although this relationship between MYBL2 and mTOR pathway has only been verified in plants, a possibility of potential correlation between them in mammals should not be ignored.

Moreover, the mTOR pathway plays a major role in the glucose metabolic program in cancers [14], and the alteration of glucose metabolism is one of the first identified hallmarks of cancer [15]. Notably, lncRNA MALAT1 regulates the mTOR pathway in various tissues and tumors [16–18], especially in hepatocellular carcinoma (HCC). It has been reported that MALAT1 regulates cancer metabolism and the Warburg effect via the mTOR pathway in HCC [16], and also regulates MYBL2 expression level in HCC by affecting the alternative splicing of its pre-mRNA [19]. However, the crosstalk between MALAT1, MYBL2, and mTOR pathway has not been reported, and the clinical significance of MALAT1 and MYBL2 requires further investigation.

In the present study, we showed that MYBL2 is a downstream target of MALAT1. MALAT1 knockdown inhibits the expression of MYBL2 and reduced the phosphorylation level of mTOR pathway. Silencing MALAT1 and MYBL2 or using mTOR pathway inhibitor rapamycin significantly blocks PCa proliferation and decreases the Warburg effect. We thus reveal the MALAT1/MYBL2/mTOR axis and that MALAT1 and MYBL2 regulate PCa glucose metabolism via the mTOR pathway.

## 2. Materials and Methods

### 2.1. Cell Culture

The human PCa cell lines, PC-3 and C4-2, and the prostate epithelial cell line RWPE-1 were purchased from the American Type Culture Collection (ATCC). PC-3 and C4-2 cells were cultured in RPMI-1640 medium (Solarbio) containing 10% fetal bovine serum (FBS, HyClone), while RWPE-1 was cultured in a keratinocyte serum-free medium (K-SFM) kit (Invitrogen). All cell lines were tested and authenticated by short tandem repeat (STR) profiling. The cells were grown in a humidified incubator under 5% CO_2_ at 37°C.

### 2.2. Patients and Tissue Specimens

This study was conducted on 98 archived paraffin-embedded clinical specimens (including 3 normal prostatic tissues and 95 primary or localized PCa tissues) that were diagnosed both clinically and pathologically (Ethic Number: HProA150PG02). The clinical samples were provided by Shanghai Outdo Biotech Company and approved by the Institutional Research Ethics Committee in Shanghai of China for research purposes and the necessary information of patients is in the supplementary materials.

### 2.3. RNA-seq Assay and Data Analysis

A minimum of 3 *μ*g of total RNA was isolated, and cDNA libraries were prepared using the Illumina TruSeq mRNA Stranded Library Prep Kit (Illumina) according to the manufacturer's protocol and analyzed by sequencing on an Illumina HiSeq 2000 sequencing platform (BerryGenomics). Reads per kilobase pair per million mapped (RPKM) reads value for each gene were assessed. A gene is considered significantly differentially expressed if the expression differs between any two samples with *P* value <0.05, as calculated by Cufflinks and compared to the potential mRNA of MALAT1 predicted by RNA RNAINTER (http://www.rnainter.org).

### 2.4. siRNA Treatment

Individual siRNAs against MALAT1 and MYBL2 are designed and provided by Genepharma Company, and the sequences of siRNAs are shown in Supplementary Table S1. The siRNAs were transfected into cells using the transfection reagent (Polyplus), following the manufacturer's instructions.

### 2.5. RT-qPCR

Total RNA was extracted using TRIzol (Thermo Fisher), and 0.5 *μ*g of total RNA was reverse transcribed using M-MLV reverse transcriptase (Promega) after DNase treatment (Promega). Subsequently, qPCR was performed on the cDNA using SYBR Green Mix (Roche) on CFX96 (Bio-Rad) real-time PCR machine. The primers are listed in Supplementary Table S1.

### 2.6. Immunoblotting

RIPA buffer was used for cell lysis. An equivalent of 15 *μ*g total protein from each cell lysate was separated by 10% SDS-PAGE and transferred to a polyvinylidene difluoride (PVDF) membrane. After blocking the membrane with 5% skimmed milk, the primary antibodies were MYBL2 (1 : 2000, Proteintech), GAPDH (1 : 5000, Preoteintech), *α*-tubulin (1 : 2500, Affinity), mTOR (1 : 2000, Preoteintech), Phospho-mTOR (1 : 2000, Proteintech), and PKM2 (1 : 2500, Affinity), and the secondary antibodies were goat antimouse and goat antirabbit (1 : 10000, Proteintech).

### 2.7. EdU Assay

Cell proliferation was estimated using the BeyoClick™ EdU cell proliferation kit (Beyotime). After transfection, .1.5 × 105 cells/well were seeded in six-well plates for 48 h and incubated with 10 *μ*M EdU at 37°C for 2 h. After fixation with 4% paraformaldehyde for 30 min, the cells were exposed to 0.1% Triton X-100 for 10 min and washed with phosphate-buffered saline. Subsequently, the cells were treated with Click-iT EdU cocktail for 30 min and stained with 5 *μ*g/mL of Hoechst 33342 for 30 min [20]. The findings were analyzed by using Olympus IX73 microscope.

### 2.8. Colony Formation Assay

Briefly, 1000 cells/well were seeded in six-well plates and cultivated for 9 days. Then, the cells were fixed with 2.5% glutaraldehyde and stained with methylene blue solution (1% methylene blue in 0.1 mol/L borate buffer, pH 8.5) at room temperature for 30 min. The plates were dried to capture the images [21].

### 2.9. Metabolite Detection and Analysis

We used wild-type and MYBL2 knockdown PC-3 cells at a density of 10^7^ cells. The supernatants were collected by centrifuging for 20 minutes (14,000*g*, 4°C). Then, 800 *μ*L precooled methanol/acetonitrile (1 : 1, v/v) was added; the mixture was sonicated in an ice bath for 20 min and incubated for −20°C 1 h for protein precipitation. Subsequently, the supernatant was collected by centrifugation of the mixture at 14,000*g* at 4°C for 20 min and analyzed on an Agilent 1290 Infinity LC system coupled with AB 5500 QTRAP mass spectrometer [22].

### 2.10. Extracellular Acidification Rate (ECAR) Assay

The capacity for glycolysis was assessed using the Seahorse XF cell Mito stress test kit or glycolysis stress test kit from Seahorse Bioscience on a Seahorse Bioscience XF96 extracellular flux analyzer (MA, USA). Cells (1.5 × 104 cells/well) were cultured in microplates XF96 (Seahorse Bioscience) the day before estimation. Finally, the ECAR values were measured by adding oligomycin (2 *μ*M), 2-deoxy-glucose (100 mM), and glucose (10 mM) to the plate. The final ECAR values were normalized to the cell numbers [23].

### 2.11. Mice

All animal experiments were approved by the Institutional Animal Care and Use Ethics Committee of Shanghai CQ and performed in the Department of Laboratory Animals of Shanghai CQ under the institutional guidelines. Adult male nude mice (*n* = 12, 6–7 weeks old) were purchased from the Shanghai CQ and raised in a barrier facility under a 12-h light/dark cycle. Mice were randomly divided into NC or siMALAT1 group with 6 mice in each group. Then, vector control or MALAT1 knockdown PC-3 cells were injected subcutaneously into the flanks of mice NC or siMALAT1 group. Next, we recorded tumor volume once a week for a total of 6 weeks. Six weeks later, mice were sacrificed, and tumors of each mice were excised, imaged, and weighed. Ki67, MYBL2, Phospho-mTOR, mTOR, and PKM2 protein levels were measured by immunohistochemistry.

### 2.12. Immunohistochemistry (IHC)

Tumor tissues were isolated from mice and PCa patients and sliced into 5-*μ*m sections. After formalin fixation and paraffin embedding, antigen retrieval was performed in citrate buffer (Vector Labs #H-3300), and the sections were blocked using 3% H2O2. Subsequently, the slides were incubated with primary antibody Ki67 (Affinity), MYBL2 (Affinity), mTOR (Proteintech), phospho-mTOR (Proteintech), and PKM2 (Affinity), and secondary antibodies antirabbit and antimouse (Proteintech). Finally, the slides were incubated with the horseradish peroxidase substrate diaminobenzidine (Thermo Scientific) and counterstained with hematoxylin, and we used IHC score to quantify IHC results.

### 2.13. Statistical Analysis

Statistical analyses were conducted using SPSS 21.0 or GraphPad Prism 7 software. The error bars represent the standard deviations from the mean. All experiments were repeated at least three times. The difference between groups was analyzed by Student unpaired *t* test. *P* values displayed as ∗*P* < 0.05, ∗∗*P* < 0.01, and ∗∗∗*P* < 0.001 indicated statistical significance.

## 3. Results

### 3.1. MALAT1 Is Upregulated in PCa Cell Lines and May Control MYBL2 mRNA Expression

**Figure 1:**
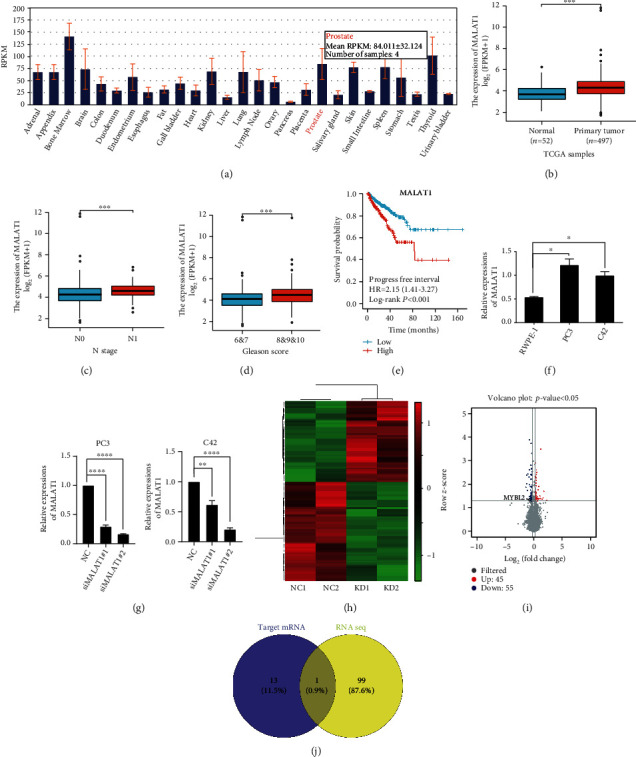
MALAT1 shows upregulated expression in PCa cell lines and may control mRNA MYBL2. (a) The expression of lncRNA MALAT1 in various tissues. (b–e) MALAT1 level assessed by analyzing TCGA database. The representative images were obtained by primary tumor (b), N stage (c), Gleason score (d), and progression-free interval (e). (f) QRT-PCR analyzed of MALAT1expression among C4-2, PC-3, and RWPE-1; GAPDH was used an internal control. (g) QRT-PCR displayed the knockdown efficiency in PC-3 and C4-2 by treating with MALAT1 siRNA. GAPDH was used an internal control. (h–i) The RNA-seq result shows all mRNA changes between MALAT1 knockdown PC-3 cell lines and control cell lines (*n* = 2). (j) Weien picture demonstrate intersection between RNA-seq result and MALAT1 potential target mRNA. The error bars indicate SD (*n* = 3). Student *t* test was used. ∗*P* < 0.05, ∗∗*P* < 0.01, ∗∗∗*P* < 0.001.

To verify the role of MALAT1 in PCa, we first analyzed the MALAT1 expression profile in primary organ tissues and PCa using the Cancer Genome Atlas (TCGA) database and found that MALAT1 was highly expressed in the prostate than other tissues, and the expression was even higher in PCa (Figures 1(a) and 1(b)). In the N1 stage, the MALAT1 RNA levels were upregulated compared to N0, which is correlated with high PSA levels and low progression-free survival rates (Figures 1(c)–1(e)). We also found that the expression of MALAT1 was significantly higher in PC-3 and C4-2 PCa cell lines than in the prostate epithelial cell line RWPE-1 (Figure 1(f)). Although associated with several aspects of cancer progression, the function and mechanism of MALAT1 in PCa need to be explored further. Thus, we used siRNA to create MALAT1 knockdown PC-3 and C4-2 cell lines (Figure 1(g)) and selected the most efficient knockdown cell clones for RNA-seq (Figures 1(h) and 1(i)) analysis. On the other hand, potential mRNA targets of MALAT1 were predicted using the RNAINTER database (Table 1 and Figure 1(j)), and we found that 14 mRNAs had a potential score >0.95. These findings were compared to the RNA-seq data, and MYBL2 mRNA was matched from both studies.

### 3.2. MALAT1 Regulates Downstream mRNA MYBL2 and Confer PCa Progression

**Figure 2:**
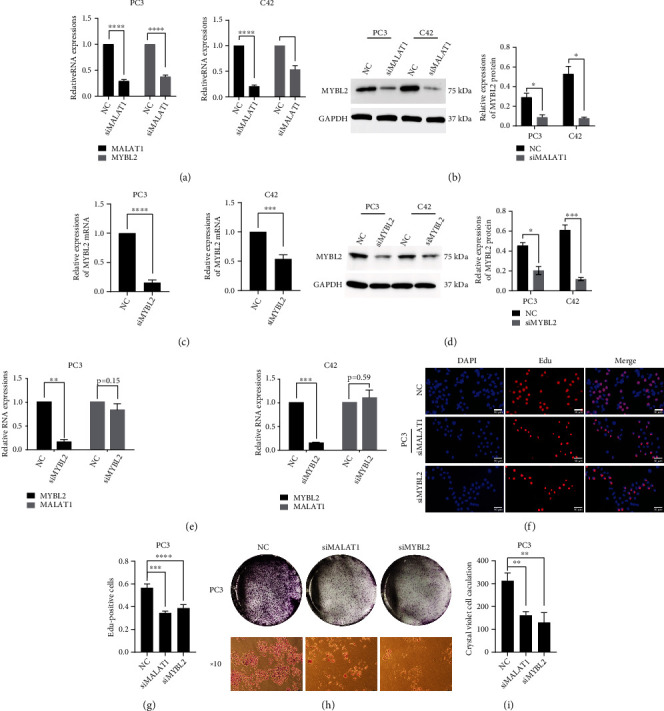
MALAT1 regulates downstream mRNA MYBL2 and confer PCa progression. (a) QRT-PCR verified the MYBL2 levels in MALAT1 knockdown PC-3 cell line (left panel) and C4-2 cell line (right panel). (b) Western blot examines MYBL2 level in MALAT1 knockdown PC-3 and C4-2 cell lines (left panel), and quantification (right panel) of western blot result. (c) QRT-PCR displayed the knockdown efficiency in PC-3 and C4-2 by treating with MYBL2 siRNA. (d) Western blot tests MYBL2 level after treating PC-3 and C4-2 cell line with siMYBL2 (left panel) and quantification (right panel) of western blot result. (e) QRT-PCR examines the MALAT1 level in knockdown MYBL2 PC-3 cell line (left panel) and in C4-2 (right panel). (f) Representative images of EdU assay formed by the vector control PC-3 cell line and MALAT1 or MYBL2 knockdown cell lines. Scale bars: 50 *μ*m. (h) Representative images of colonies formed by vector control PC-3 cell line and MALAT1 or MYBL2 knockdown cell lines. (g and i) Quantification of EdU assay (left panel) and colonies (right panel). All samples were normalized to GAPDH mRNA and protein levels. The error bars indicate SD (*n* = 3). Student *t* test was used. ∗*P* < 0.05, ∗∗*P* < 0.01, ∗∗∗*P* < 0.001.

Next, we tried to verify the regulatory correlation between MALAT1 and MYBL2 and found that MALAT1 knockdown decreased the expression of MYBL2 at both RNA and protein levels (Figures 2(a) and 2(b)). In contrast, transient knockdown of MYBL2 by siRNA in PC-3 and C4-2 cell lines (Figures 2(c) and 2(d)) did not show significant changes in MALAT1 expression, indicating that MYBL2 is a gene downstream of MALAT1 (Figure 2(e)). Reportedly, MALAT1 regulates MYBL2 through the splicing factor SRSF1 in HCC [19]. We first examined the possible mechanism of MALAT1regulating MYBL2 in PCa. In MALAT1 knockdown cell lines, SRSF1 was associated with MYBL2 spliced exon (Figure S1E and F), indicating MALAT1 might regulate MYBL2 via the binding of SRSF1 to MYBL2 pre-mRNAs. Next, we investigated the function of MALAT1 and MYBL2 in PCa progression. In the EdU assay, the cells were fluorescent-labeled in 2 h, representing the PCa proliferation capacity. Silencing MALAT1 or MYBL2 by siRNAs inhibited the generation of new cells in both knockdown cell lines (Figures 2(f) and S1A). Furthermore, MALAT1- or MYBL2-downregulated PCa cells formed fewer and smaller colonies than the vector control in the colony formation assay (Figures 2(h) and S1B). These results suggested that MALAT1 regulates MYBL2 in PCa and mediates PCa proliferation capacity.

### 3.3. Silencing MALAT1 or MYBL2 Suppresses the Expression of Phosphorylated mTOR (p-mTOR)

**Figure 3:**
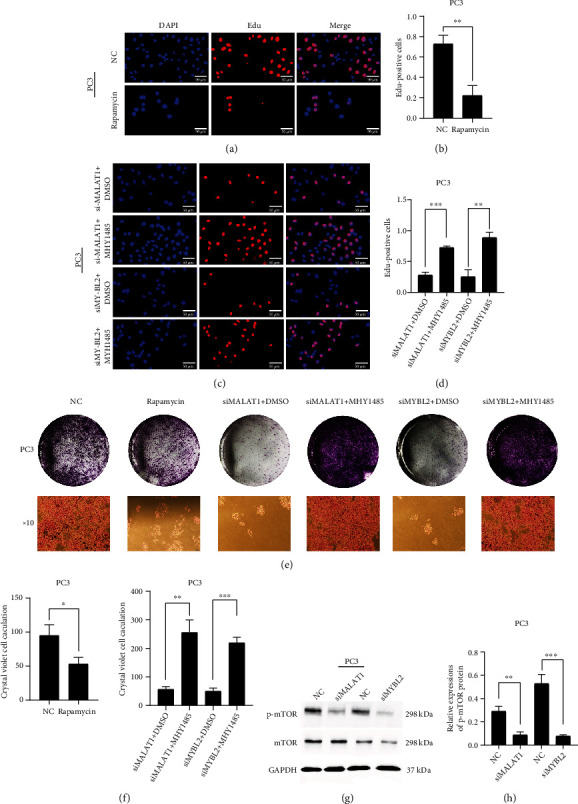
Silencing MALAT1 or MYBL2 suppresses the expression of phospho-mTOR. Representative images (a) and quantification (b) of EdU assay formed by PC-3 cell line treated with vector control or mTOR pathway inhibitor rapamycin. Scale bars: 50 *μ*m. Representative images (c) and quantification (d) of EdU assay formed by MALAT1 or MYBL2 knockdown PC-3 cell line and those cell lines treated with mTOR pathway activator MHY1485. Scale bars: 50 *μ*m. (e) Representative images of colonies formed by vector control PC-3 cell line, added rapamycin PC-3 cell line, MALAT1 or MYBL2 knockdown PC-3 cell line, and those two cell lines treated with MHY1485. (f) Quantification of colonies. Western blot examines mTOR and phospho-mTOR (p-mTOR) level in MALAT1 or MYBL2 knockdown PC-3 cell line (g), and quantification (h) of western blot result. All samples were normalized to GAPDH mRNA and protein levels. The error bars indicate SD (*n* = 3). Student *t* test was used. ∗*P* < 0.05, ∗∗*P* < 0.01, ∗∗∗*P* < 0.001.

mTOR pathway plays a critical role in tumorigenesis and cancer progression [12], and a potential correlation has been identified between MYBL2 and mTOR in *Arabidopsis* [13]. In order to explore whether MALAT1 and MYBL2 are related to the mTOR pathway in PCa, we utilized mTOR pathway inhibitor rapamycin and found that it reduced the growth rate (Figures 3(a) and S1G) and the number of colonies of PCa cell lines significantly (Figures 3(b) and S1H). MALAT1 or MYBL2 knockdown cell lines were then treated with mTOR activator MHY1485 to assess the differences in the proliferation ability of the cells. Compared to the original MALAT1 or MYBL2 knockdown cell lines, MHY1485 addition restored the cell growth capacity, assessed by the EdU assay (Figures 3(b) and S1). A similar effect was noted in the colony formation assay: knockdown cell lines treated with MHY1485 generated larger colonies than the MALAT1 or MYBL2 knockdown cell lines (Figures 3(e) and S1K). This accumulating evidence supported our hypothesis that both MALAT1 and MYBL2 regulate the mTOR pathway. For further verification, we examined the protein level of mTOR. Western blot data did not show any difference in the protein expression between MALAT1 or MYBL2 knockdown and control cell lines. We thus explored the expression of p-mTOR and found that it was inhibited significantly after MALAT1 or MYBL2 knocking down (Figures 3(h) and S2B). These findings suggested that MALAT1 regulates MYBL2 and controls the phosphorylation level of the mTOR pathway in PCa cells.

### 3.4. MALAT1/MYBL2/mTOR Axis Mediates PCa Glucose Metabolism

**Figure 4:**
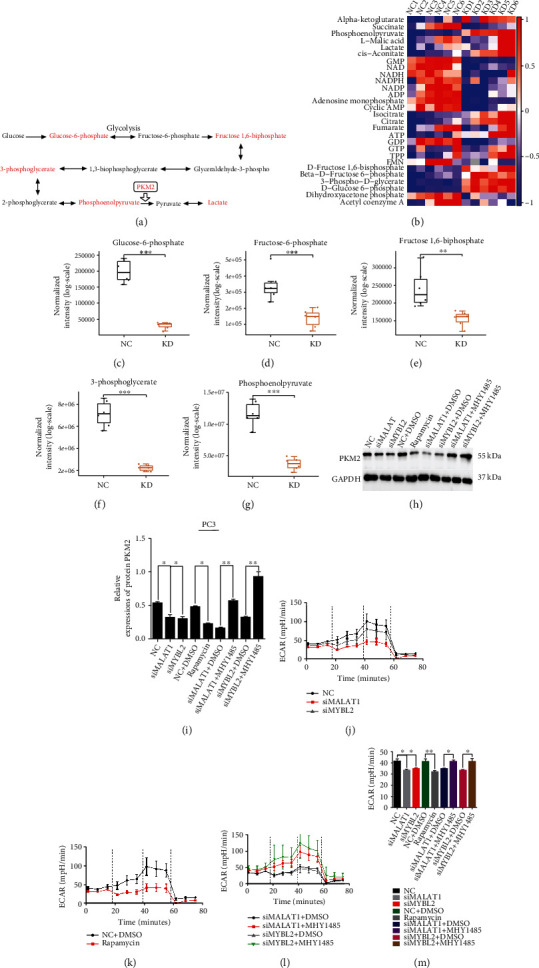
MALAT1/MYBL2/mTOR axis mediates PCa glucose metabolism. (a) Schematic representation of the glycolytic pathways of glucose metabolism. The enzymes marked in red were selected for targeted metabolomics analysis. (b–g) The results of targeted metabolomics (*n* = 6) analysis revealed that silencing MYBL2 in PCa leads to significantly decreasing of glucose-6-phosphate (c), fructose-6-phosphate (d), fructose-1,6-biophosphate (e), 3-phosphoglycerate (f), and phosphoenolpyrucate (g). Western blot examines PKM2 level in all mentioned PC-3 cell lines (h) and quantification (i) of western blot result. (j–m) Extracellular acidification rate (ECAR) assay. ECAR among vector control and MALAT1 or MYBL2 knockdown PC-3 cell lines (j), ECAR between vector control and added rapamycin PC-3 cell lines (k), ECAR among MALAT1 or MYBL2 knockdown PC-3 cell lines and those cell lines treated with MHY1485 (l), and overall lactate level of all PC-3 cell lines mentioned before (m). All samples were normalized to GAPDH mRNA and protein levels. The error bars indicate SD (*n* = 3). Student *t* test was used. ∗*P* < 0.05, ∗∗*P* < 0.01, ∗∗∗*P* < 0.001.

The increased understanding of cancer has identified glucose metabolism as one of the first hallmarks of cancer [24]. The mTOR pathway affects tumor metabolism through multiple approaches [25]. Therefore, we investigated the effect of the MALAT1/MYBL2/mTOR axis on glucose metabolism in PCa cells. We examined the products of metabolism which were compared between MYBL2 knockdown and control cell lines through targeted metabolomics analysis. The results showed that the levels of products of glycolysis declined in MYBL2 knockdown cell lines (Figures 4(a)–4(g)). These findings provided strong evidence that the MALAT1/MYBL2/mTOR axis participates in tumor glycolysis. The M2 isoform of the glycolytic enzyme pyruvate kinase (PKM2) is the preferred splice isoform of pyruvate kinase in cancer cells that converts phosphoenolpyruvate to pyruvate, which is critical for aerobic glycolysis in cancer cells [26]. We thus investigated the protein level of PKM2 in the MALAT1/MYBL2/mTOR axis and found that silencing MALAT1, MYBL2, or inhibited mTOR by using rapamycin decreases the expression of PKM2 in PCa cell lines, and the addition of the mTOR pathway activator could reverse this phenomenon (Figures 4(h) and S2D). Interestingly, PKM2 plays a critical role in the Warburg effect, a unique metabolic characteristic of tumor tissues and tumor development [27]. In order to verify whether MALAT1/MYBL2/mTOR affects the Warburg effect in PCa, we utilized extracelluar acidification rate (ECAR) to display the glycolysis capacity of the PCa cell lines. Silencing the MALAT1/MYBL2/mTOR axis decreased the glycolytic flux and the overall lactate level in PCa cells (Figures 4(j)–4(m) and S2F–I). Collectively, our results showed that the MALAT1/MYBL2/mTOR axis regulates glucose metabolism and plays a role in the Warburg effect in PCa cells.

### 3.5. MALAT1/MYBL2/mTOR Axis Regulates PCa Progression and Glucose Metabolism in the Mouse Model

**Figure 5:**
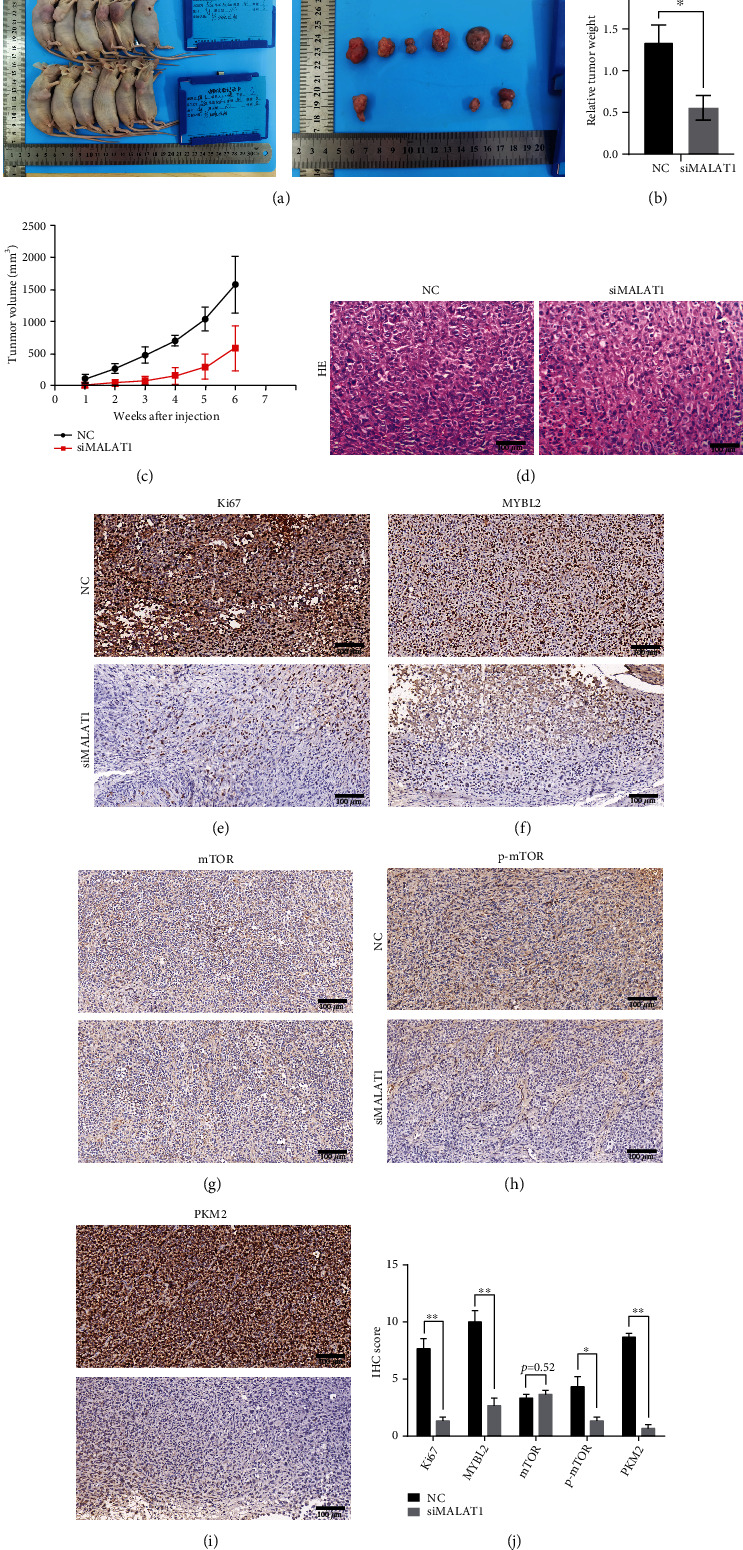
MALAT1/MYBL2/mTOR axis regulates PCa progression and glucose metabolism in mouse model. (a) The tumorigenic ability of nude mouse model (*n* = 6) between injected knockdown MALAT1 PC-3 cell siMALAT1 group and vector control NC group. (b) Final tumor weight between siMALAT1 group and NC group. (c) Tumor volume growth between two groups. (d) Representative images of hematoxylin-eosin staining between two groups. Scale bars: 100 *μ*m. (e–i) Representative images of immunohistochemistry of Ki67 (e), MYBL2 (f), mTOR (g), p-mTOR (h), and PKM2 (i) between siMALAT1 group and NC group. (j) Quantification of IHC results. Scale bars: 100 *μ*m. The error bars indicate SD (*n* = 6). Student *t* test was used. ∗*P* < 0.05, ∗∗*P* < 0.01.

The MALAT1/MYBL2/mTOR axis was then evaluated in the mouse model. In the in vitro experiment, silencing the MALAT1/MYBL2/mTOR axis significantly reduced colony number and glycolysis. Next, we injected MALAT1 knockdown PC-3 cells (siMALAT1) and vector control PC-3 cells (NC) in the mouse model, and the tumorigenic ability in the siMALAT1 group was also inhibited (Figure 5(a)): only 3/6 nude mice exhibited tumorigenicity. In addition, the tumor grew slowly in the siMALAT1 group (Figure 5(c)), and the final tumor weight indicated that the average in siMALAT1 was lower than that in the NC group (Figure 5(b)). Besides, we examined the expression of MALAT1 in mouse tumor (Figure S2J). We also verified the expressions of primary proteins in vitro and found that cell proliferation antigen Ki-67, MYBL2, p-mTOR, and PKM2 declined in the siMALAT1 group (Figures 5(d)–5(i)), while the mTOR protein level did not differ between the two groups (Figure 5(j)). The in vivo study further proved the functions of the MALAT1/MYBL2/mTOR axis in cancer proliferation and glucose metabolism in PCa cells.

### 3.6. Clinical Relevance of the MYBL2 in Human PCa

**Figure 6:**
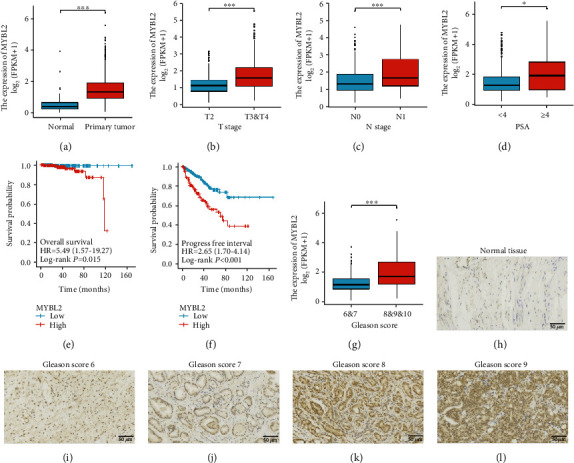
Clinical relevance of the MYBL2 in human PCa. (a–g) MYBL2 level assessed by analyzing TCGA database. The representative images were obtained by primary tumor (a), T stage (b), N stage (c), PSA (d), overall survival (e), progression-free interval (f), and Gleason score (g). (h–l) Representative images of normal prostate tissues (h), Gleason score 6 (i), Gleason score 7 (j), Gleason score 8 (k), and Gleason score 9 (l) staining in 95 prostate cancer patients and 3 normal human prostate specimens. Scale bars: 50 *μ*m.

**Figure 7:**
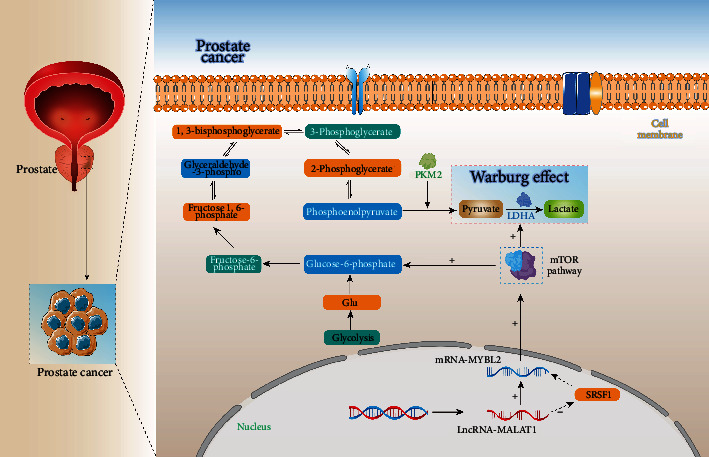
Model: MALAT1 regulates MYBL2 and mediates mTOR pathway in PCa, MALAT1/MYBL2/mTOR plays an important role in glucose metabolism and may participate in Warburg effect in prostate cancer.

The clinical relevance of MYBL2, a potential carcinogen, was evaluated in human PCa. First, we analyzed TCGA data and found that MYBL2 mRNA levels were upregulated in T3-4 stage than in T2, in N1 PCa compared to N0, and had high PSA level and Gleason score (Figures 6(a)–6(d) and 6(g)). Moreover, high expression of MYBL2 leads to a poor overall survival rate (*P* = 0.015) and lower progression-free survival (*P* < 0.001) (Figures 6(e) and 6(f)). We also examined the specific correlation between MYBL2 and Gleason score in human PCa specimens. Compared to the normal prostate tissue, the MYBL2 level was higher in patients with Gleason scores 6 and 7, and the expression was significantly advanced in those with Gleason scores 8 and 9 (Figures 6(h)–6(l)). These results support the idea that MYBL2 is a potential oncogene in PCa and that the MALAT1/MYBL2/mTOR axis plays a role in PCa progression (Figure 7).

## 4. Discussion

LncRNA MALAT1 is a classical carcinogen in various tumors, controlling several aspects of cancer progression [4]. In PCa, MALAT1 is associated with tumor enhancing to CRPC [28]. A recent study revealed that MALAT1 also acts as a tumor suppressor gene in breast cancer [29]. These controversial characteristics have prompted an intensive focus on MALAT1 and its functions and mechanisms in PCa. Therefore, we analyzed the MALAT1 data using the TCGA database and found that MALAT1 is associated with PCa metastasis, Gleason score, and progression-free interval rate. We thus utilized RNA-seq and RNAINTER platforms and identified MYBL2 mRNA as the highly potential target of MALAT1 matching the standard.

As a potential oncogene and biomarker in aggressive cancers [30, 31], MYBL2 stimulates the malignant progression of tumors via cancer proliferation, drug resistance, and metastasis [32, 33]. Recently, MYBL2 has also been shown to play a critical role in CRPC progression [11, 34, 35]. TCGA database analysis revealed that MYBL2 is linked to PCa metastasis, Gleason score, and survival rate. These functions of MYBL2 support our finding that MALAT1 regulates MYBL2 in PCa, which was further verified at RNA and protein levels. Also, MYBL2 knockdown confirmed it as a downstream target gene of MALAT1 in PCa. The specific functions of MALAT1 and MYBL2 in PCa revealed in the present study were consistent with the previous reports. Interestingly, MALAT1 regulates MYBL2 by controlling the association of SRSF1 to pre-mRNA MYBL2 exons [19]. We observed similar results in MALAT1 knockdown PCa cell lines. However, the mechanism underlying MALAT1-regulation of MYBL2 needs further exploration.

mTOR signaling pathway affects tumor proliferation, metastasis, and drug resistance [24, 36], and MALAT1 mediates the mTOR pathway in multiple cancers [16, 18, 37]. In previous study, MYBL2 was a substrate for mTOR downstream GSK3-like kinase in *Arabidopsis* [38], and the mTOR pathway was shown to inhibit the activated MYBL2. Our results on the functions of MYBL2 and mTOR pathway in malignancy progress now provided the first evidence on the crosstalk between MYBL2 and mTOR pathway in mammals, as well as the role of the MALAT1/MYBL2/mTOR axis in PCa, where MYBL2 promotes the mTOR pathway in PCa cells. Nevertheless, the molecular mechanism of the MYBL2-regulated mTOR pathway requires further investigation.

Glucose metabolism is an intrinsic characteristic of cancer cells during carcinogenesis and tumor progression, proving advantageous to tumor proliferation [39]. In PCa, the hyperactivity of glucose metabolism advances the cancer stage, while inhibition of PCa glycolysis results in intracellular acidification and apoptosis, as observed in the in vitro experiments [40]. mTOR pathway plays a critical role in glucose metabolism and is a potential therapeutic target [41]. Therefore, we explored the role of the MALAT1/MYBL2/mTOR axis in glucose metabolism and found that this axis affects many products of glycolysis in PCa cells. Furthermore, PKM2 is a downstream target of the mTOR pathway: silencing the axis inhibits the PKM2 protein level markedly. PKM2 is not only a critical gene in cancer cell development and survival [42], but its role in aerobic glycolysis prompts further exploration of the function of the axis in PCa.

Aerobic glycolysis, or the “Warburg effect,” is a unique tumor metabolism promoting cancer progression [43]. However, the underlying mechanism of the Warburg effect in many tumors remains unknown. The current study revealed that the MALAT1/MYBL2/mTOR axis affects glycolysis and PKM2 level, thereby leading us to examine the correlation with the Warburg effect. The ECAR assay demonstrated the association between glycolysis capacity and MALAT/MYBL2/mTOR axis: this axis promotes overall lactate in PCa cells. Together, these findings suggested that the MALAT1/MYBL2/mTOR axis mediates the Warburg effect in PCa cells. Although there are some limitations, such as lack of oxygen consumption rate (OCR) assay to measure oxygen consumption in our verification experiments, the results clearly link the mTOR pathway with the Warburg effect.

We also verified our findings in a nude mouse model. Our study shows that the tumorigenic capacity of mice injected MALAT1 knockdown PC-3 cells was weaker than that of the control group, and only 3/6 mice progressed to tumorigenesis. This result indicated low tumorigenic capacity due to MALAT1 reduction, which was supported by the final weight and volume of the tumor growth. Overall, the results of animal experiments are consistent with the above in vitro findings.

In the present study, we revealed the metabolism function of the MALAT1/MYBL2/mTOR axis in PCa. However, since it is a novel axis, the mechanisms of its abilities require further examination. mTOR pathway is also a mediator for other metabolisms in various cancers [44], and fatty acid [45], cholesterol [46], one-carbon [47], and amino acid metabolisms [48] are gaining attention in PCa research. These previous studies prompted us to explore the roles of the MALAT1/MYBL2/mTOR axis in other metabolisms, such as lipid metabolism, because the mTOR pathway is involved in the conversion of white adipose tissue and brown adipose tissue [49]. Additionally, we explored the functions of the MALAT1/MYBL2/mTOR axis, but the regulatory mechanisms are yet to be elucidated. The mechanism between MALAT1 and MYBL2 might be effectuated through SRSF1 but needs an in-depth investigation between MYBL2 and mTOR pathway. In *Arabidopsis*, MYBL2 is a downstream target of the mTOR pathway but an upstream gene in mammals; these differences necessitate the exploration of the connection between MYBL2 and all the known upstream targets of mTOR and the underlying mechanisms. Besides, our founding reveals that MYBL2, as a highly potential tumorigene, provides a new character of MALAT1in PCa. Interestingly, more and more researches using Chinese medicine to treat cancer [50, 51] give us a new research interest in tumor treatment.

Taken together, the current study demonstrated that MALAT1 regulates MYBL2 and controls the mTOR pathway in PCa cells. We revealed MALAT1/MYBL2/mTOR axis for the first time and suggested that this novel axis mediates glycolysis and the Warburg effect in PCa cells. These results pointed out that silencing MALAT/MYBL2/mTOR axis might be considered as potential new therapy based on glucose metabolism for PCa .

## Figures and Tables

**Table 1:** Potential targeted mRNA of MALAT1.

Interactor1	Category1 species(homo)	Interactor2	Category2 species(homo)	Score
MALAT1	lncRNA	SMAD3	mRNA	0.9988
MALAT1	lncRNA	MARCH7	mRNA	0.9975
MALAT1	lncRNA	ZEB1	mRNA	0.982
MALAT1	lncRNA	TF	mRNA	0.9526
MALAT1	lncRNA	ZEB2	mRNA	0.9526
MALAT1	lncRNA	SNAI2	mRNA	0.9526
MALAT1	lncRNA	MMP9	mRNA	0.9526
MALAT1	lncRNA	TP53	mRNA	0.9418
MALAT1	lncRNA	PTBP3	mRNA	0.9418
MALAT1	lncRNA	PIK3CB	mRNA	0.9418
MALAT1	lncRNA	MYBL2	mRNA	0.9418
MALAT1	lncRNA	MMP2	mRNA	0.9418
MALAT1	lncRNA	MMP14	mRNA	0.9418
MALAT1	lncRNA	MAPK8	mRNA	0.9418

## Data Availability

We declare that the materials described in the manuscript, including all relevant raw data, will be freely available to any scientist wishing to use them for noncommercial purposes, without breaching participant confidentiality.

## Supplementary Materials

Supplementary 1 Figure S1. (A) Representative images of EdU assay formed by the vector control C4-2 cell line and MALAT1 or MYBL2 knockdown cell lines. Scale bars: 50 *μ*m. (B) Representative images of colonies formed by vector control C4-2 cell line and MALAT1 or MYBL2 knockdown cell lines. (C and D) Quantification of EdU assay (left panel) and colonies (right panel). (E, F) QRT-PCR analyses of SRSF1 RNAIP samples from control and MALAT1 knockdown PC-3 and C4-2 cell lines reveal increased association of SRSF1 to MYBL2 mRNA. (G and I) Representative images (left panel) and quantification (right panel) of EdU assay formed by C4-2 cell line treated with vector control or mTOR pathway inhibitor rapamycin. Scale bars: 50 *μ*m. (H and J) Representative images (left panel) and quantification (right panel) of EdU assay formed by MALAT1 or MYBL2 knockdown C4-2 cell line and those cell lines treated with mTOR pathway activator MHY1485. Scale bars: 50 *μ*m. (K) Representative images of colonies formed by vector control C4-2 cell line, added rapamycin PC-3 cell line, MALAT1 or MYBL2 knockdown C4-2 cell line, and those two cell lines treated with MHY1485.

Supplementary 2 Figure S2. (A) Quantification of colonies. (B and C) Western blot examines mTOR and phospho-mTOR (p-mTOR) level in MALAT1 or MYBL2 knockdownC4-2 cell line (left panel) and quantification (right panel) of western blot result. (D and E) Western blot examines PKM2 level in all mentioned C4-2 cell lines (left panel), and quantification (right panel) of western blot result. (F–I) Extracellular acidification rate (ECAR) assay. ECAR among vector control and MALAT1 or MYBL2 knockdown C4-2 cell lines (F), ECAR between vector control and added rapamycin C4-2 cell lines (G), ECAR among MALAT1 or MYBL2 knockdown C4-2 cell lines and those cell lines treated with MHY1485 (H), and overall lactate level of all C4-2 cell lines mentioned before (I). (J) Relative expression of MALAT1 in the tumor excised from mice. All samples were normalized to GAPDH mRNA and protein levels. The error bars indicate SD (*n* = 3). Student *t* test was used. ∗*P* < 0.05, ∗∗*P* < 0.01, ∗∗∗*P* < 0.001.

Supplementary 3 Table S1. List of qRT-PCR primers and siRNAs sequences.

Supplementary 4 Ethics Materials. The necessary information of prostate cancer patients.
