# Supplementary figures and images for: LncRNA-MALAT1 Regulates Cancer Glucose Metabolism in Prostate Cancer via MYBL2/mTOR Axis
Source: Oxid Med Cell Longev. 2022 May 2;2022:8693259. doi: 10.1155/2022/8693259 (PMC9086835; doi:10.1155/2022/8693259)

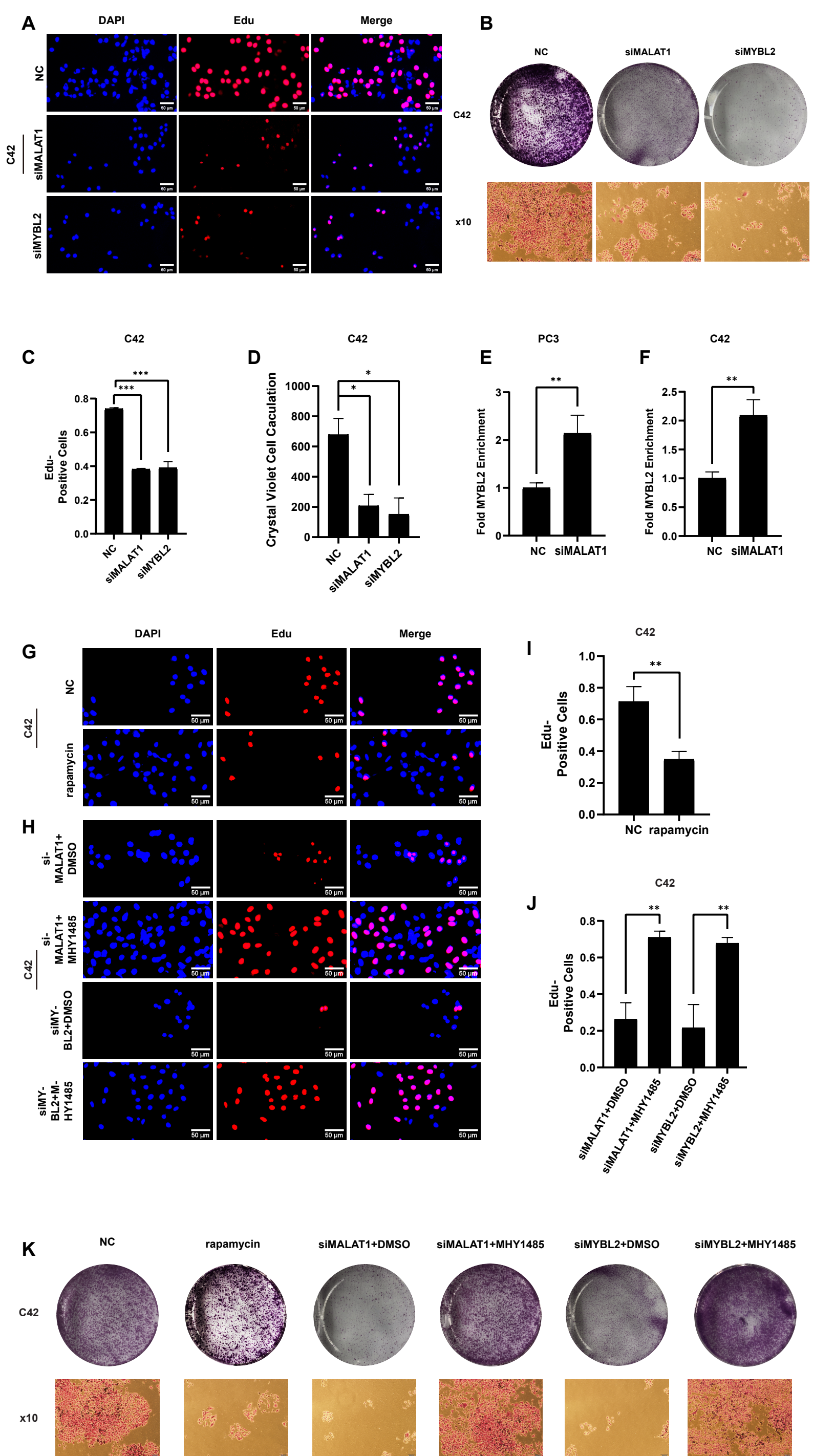

Supplement: Supplementary 1 — Figure S1. (A) Representative images of EdU assay formed by the vector control C4-2 cell line and MALAT1 or MYBL2 knockdown cell lines. Scale bars: 50 μm. (B) Representative images of colonies formed by vector control C4-2 cell line and MALAT1 or MYBL2 knockdown cell lines. (C and D) Quantification of EdU assay (left panel) and colonies (right panel). (E, F) QRT-PCR analyses of SRSF1 RNAIP samples from control and MALAT1 knockdown PC-3 and C4-2 cell lines reveal increased association of SRSF1 to MYBL2 mRNA. (G and I) Representative images (left panel) and quantification (right panel) of EdU assay formed by C4-2 cell line treated with vector control or mTOR pathway inhibitor rapamycin. Scale bars: 50 μm. (H and J) Representative images (left panel) and quantification (right panel) of EdU assay formed by MALAT1 or MYBL2 knockdown C4-2 cell line and those cell lines treated with mTOR pathway activator MHY1485. Scale bars: 50 μm. (K) Representative images of colonies formed by vector control C4-2 cell line, added rapamycin PC-3 cell line, MALAT1 or MYBL2 knockdown C4-2 cell line, and those two cell lines treated with MHY1485. [file 8693259.f1.pdf]

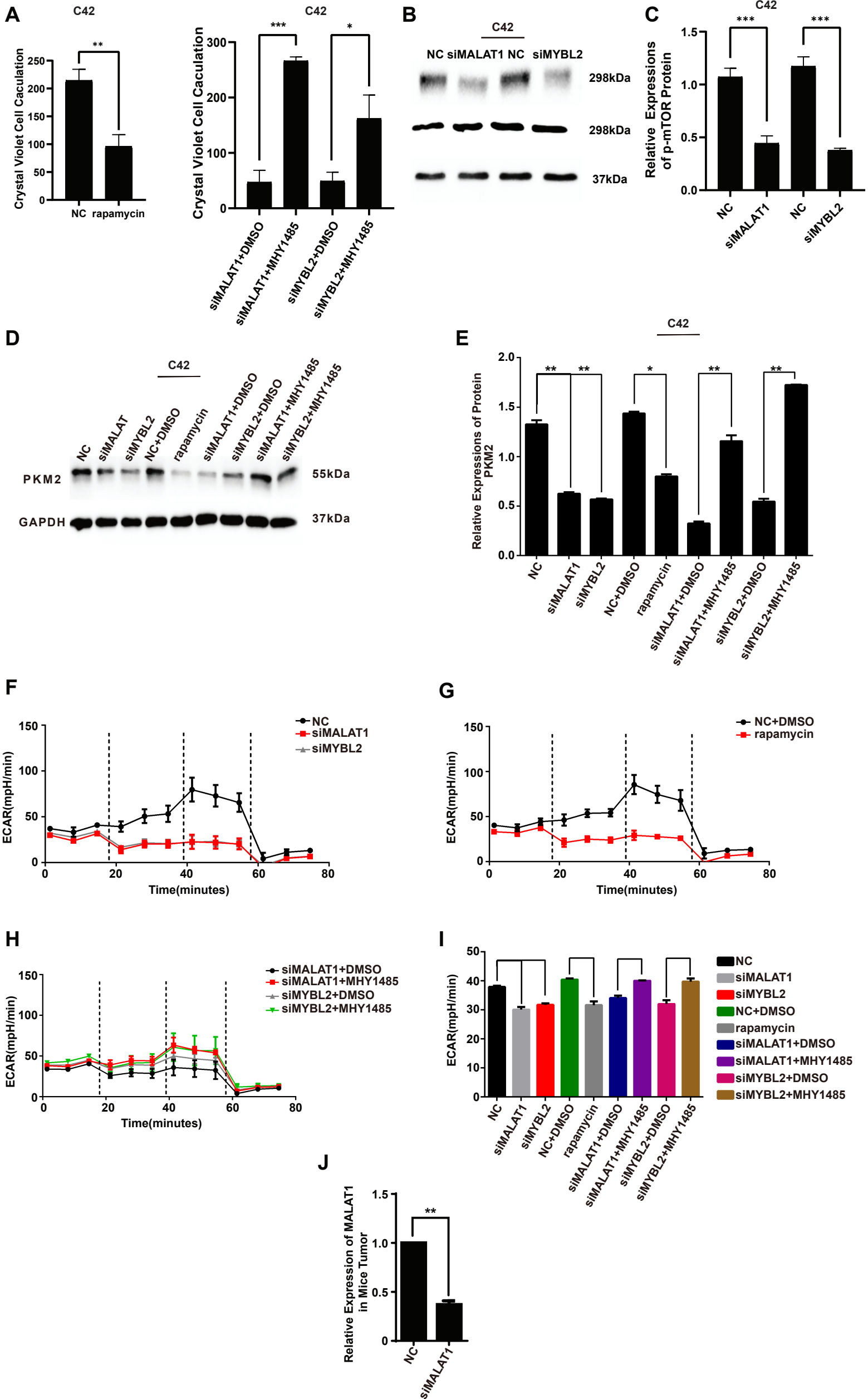

Supplement: Supplementary 2 — Figure S2. (A) Quantification of colonies. (B and C) Western blot examines mTOR and phospho-mTOR (p-mTOR) level in MALAT1 or MYBL2 knockdownC4-2 cell line (left panel) and quantification (right panel) of western blot result. (D and E) Western blot examines PKM2 level in all mentioned C4-2 cell lines (left panel), and quantification (right panel) of western blot result. (F–I) Extracellular acidification rate (ECAR) assay. ECAR among vector control and MALAT1 or MYBL2 knockdown C4-2 cell lines (F), ECAR between vector control and added rapamycin C4-2 cell lines (G), ECAR among MALAT1 or MYBL2 knockdown C4-2 cell lines and those cell lines treated with MHY1485 (H), and overall lactate level of all C4-2 cell lines mentioned before (I). (J) Relative expression of MALAT1 in the tumor excised from mice. All samples were normalized to GAPDH mRNA and protein levels. The error bars indicate SD (n = 3). Student t test was used. ∗P < 0.05, ∗∗P < 0.01, ∗∗∗P < 0.001. [file 8693259.f2.pdf]
