# Supplementary material for: LncRNA-MALAT1 Regulates Cancer Glucose Metabolism in Prostate Cancer via MYBL2/mTOR Axis
Source: Oxid Med Cell Longev. 2022 May 2;2022:8693259. doi: 10.1155/2022/8693259 (PMC9086835; doi:10.1155/2022/8693259)
Supplement: Supplementary 3 — Table S1. List of qRT-PCR primers and siRNAs sequences. [file 8693259.f3.docx]

| Sequences of primers | |
| --- | --- |
| MALAT1-Homo-F | GACGGAGGTTGAGATGAAGC |
| MALAT1-Homo-R | ATTCGGGGCTCTGTAGTCCT |
| MYBL2-Homo-F | CTTGAGCGAGTCCAAAGACTG |
| MYBL2-Homo-R | AGTTGGTCAGAAGACTTCCCT |
| MYBL2-exon-F | GGAAGTCTTCTGACCAACTGGC |
| MYBL2-exon-R | GACCTGCTGACACGCTGAC |
| GAPDH-Homo-F | AAGGTGAAGGTCGGAGTCAA |
| GAPDH-Homo-R | AATGAAGGGGTCATTGATGG |
| Sequences of siRNAs | |
| siMALAT1#1 | GGGCUUCUCUUAACAUUUATT  UAAAUGUUAAGAGAAGCCCTT |
| siMALAT1#2 | GGAGGUAACAGCACAAUAUTT  AUAUUGUGCUGUUACCUCCTT |
| siMYBL2#1 | CCGUCCCUCCUACCAUAAATT  UUUAUGGUAGGAGGGACGGTT |
| siMYBL2#2 | GCCAUGGACCAAAGAGGAATT  UUCCUCUUUGGUCCAUGGCTT |
