# Supplementary material for: LncRNA-MALAT1 Regulates Cancer Glucose Metabolism in Prostate Cancer via MYBL2/mTOR Axis
Source: Oxid Med Cell Longev. 2022 May 2;2022:8693259. doi: 10.1155/2022/8693259 (PMC9086835; doi:10.1155/2022/8693259)
Supplement: Supplementary 4 — Ethics Materials. The necessary information of prostate cancer patients. [file 8693259.f4.pdf]

tissue code	age	Pathological classification	Gleason score	Grade
N0004	26	normal tissue	——	——
N0005	37	normal tissue	——	——
N0007	22	normal tissue	——	——
1908436	68	prostate cancer	3+3=6	1
1802936	68	prostate cancer	3+3=6	1
1813397	75	prostate cancer	3+3=6	1
1823359	61	prostate cancer	3+3=6	1
1836948	72	prostate cancer	3+3=6	1
1846721	74	prostate cancer	3+3=6	1
1723212	77	prostate cancer	3+3=6	1
1723691	68	prostate cancer	3+3=6	1
1727182	72	prostate cancer	3+3=6	1
1730270	61	prostate cancer	3+3=6	1
1733893	73	prostate cancer	3+3=6	1
1740043	67	prostate cancer	3+3=6	1
1615512	70	prostate cancer	3+3=6	1
1628829	71	prostate cancer	3+3=6	1
1516571	71	prostate cancer	3+3=6	1
1539350	62	prostate cancer	3+3=6	1
1910837	79	prostate cancer	3+4=7	2
1914495	69	prostate cancer	3+4=7	2
1916089	71	prostate cancer	3+4=7	2
1804909	72	prostate cancer	3+4=7	2
1811465	73	prostate cancer	3+4=7	2
1815789	71	prostate cancer	3+4=7	2
1839870	64	prostate cancer	3+4=7	2
1840448	77	prostate cancer	3+4=7	2
1852168	66	prostate cancer	3+4=7	2
1852684	60	prostate cancer	3+4=7	2
1713465	70	prostate cancer	3+4=7	2
1727786	78	prostate cancer	3+4=7	2
1737301	75	prostate cancer	3+4=7	2
1633604	73	prostate cancer	3+4=7	2
1639669	66	prostate cancer	3+4=7	2
1501727	77	prostate cancer	3+4=7	2
1508633	64	prostate cancer	3+4=7	2
1520367	70	prostate cancer	3+4=7	2
1524855	70	prostate cancer	3+4=7	2
1533208	70	prostate cancer	3+4=7	2
1410310	67	prostate cancer	3+4=7	2
1432599	76	prostate cancer	3+4=7	2
1515092	67	prostate cancer	3+4=7	2
1441940	69	prostate cancer	3+4=7	2
1912145	75	prostate cancer	4+3=7	3
1920318	84	prostate cancer	4+3=7	3
1802149	68	prostate cancer	4+3=7	3
1818226	71	prostate cancer	4+3=7	3
1745769	80	prostate cancer	4+3=7	3
1615356	80	prostate cancer	4+3=7	3
1647300	71	prostate cancer	4+3=7	3
1536073	51	prostate cancer	4+3=7	3
1539564	62	prostate cancer	4+3=7	3

1433265	70	prostate cancer	4+3=7	3
1713464	77	prostate cancer	3+5=8	——
1828932	62	prostate cancer	4+4=8	4
1920890	71	prostate cancer	3+3=6	1
1844357	63	prostate cancer	3+3=6	1
1700756	78	prostate cancer	3+3=6	1
1618202	67	prostate cancer	3+3=6	1
1917990	71	prostate cancer	3+4=7	2
1814623	61	prostate cancer	3+4=7	2
1842475	72	prostate cancer	3+4=7	2
1705186	75	prostate cancer	3+4=7	2
1728301	71	prostate cancer	3+4=7	2
1745770	76	prostate cancer	3+4=7	2
1615650	74	prostate cancer	3+4=7	2
1625579	61	prostate cancer	3+4=7	2
1629712	72	prostate cancer	3+4=7	2
1502563	72	prostate cancer	3+4=7	2
1512698	63	prostate cancer	3+4=7	2
1528164	64	prostate cancer	3+4=7	2
1536839	69	prostate cancer	3+4=7	2
1544447	75	prostate cancer	3+4=7	2
1410176	55	prostate cancer	3+4=7	2
1437918	63	prostate cancer	3+4=7	2
1914995	77	prostate cancer	4+3=7	3
1808720	80	prostate cancer	4+3=7	3
1816018	78	prostate cancer	4+3=7	3
1818016	70	prostate cancer	4+3=7	3
1842452	73	prostate cancer	4+3=7,	3
1721399	76	prostate cancer	4+3=7	3
1616888	76	prostate cancer	4+3=7	3
1620632	72	prostate cancer	4+3=7	3
1641922	70	prostate cancer	4+3=7	3
1642711	75	prostate cancer	4+3=7	3
1846438	66	prostate cancer	3+5=8	——
1538205	67	prostate cancer	3+5=8	——
1918632	73	prostate cancer	4+4=8	4
1700610	81	prostate cancer	4+4=8	4
1727665	79	prostate cancer	4+4=8	4
1636925	61	prostate cancer	4+4=8	4
1437008	76	prostate cancer	4+4=8	4
1720874	75	prostate cancer	5+3=8	——
1918590	82	prostate cancer	4+5=9	5
1714441	82	prostate cancer	4+5=9	5
1620350	72	prostate cancer	4+5=9	5
1416277	64	prostate cancer	5+4=9	5
1418474	67	prostate cancer	5+4=9	5
